# Characterization and application of tannase and gallic acid produced by co-fungi of *Aspergillus niger* and *Trichoderma viride* utilizing agro-residues substrates

**DOI:** 10.1038/s41598-023-43955-5

**Published:** 2023-10-05

**Authors:** Alshaymaa I. Ahmed, Khadiga A. A. Abou-Taleb, Basma T. Abd-Elhalim

**Affiliations:** 1 Department of Agricultural Microbiology, Faculty of Agriculture, Beni-Suef University, Beni-Suef, Egypt; 2 Agricultural Microbiology Department, Faculty of Agriculture, Ain Shams University, Hadayek Shubra, Cairo, 11241 Egypt

**Keywords:** Industrial microbiology, Biotechnology, Microbiology

## Abstract

Bioconversion using fungi, as natural factory of many applicable bioactive compounds, as enzymes utilizing agro-residue substrates as a solid, abundant, low-cost growth and enzyme production media. This study characterized and applied a tannase enzyme (308 U/mg) from *Aspergillus niger* A8 + *Trichoderma viride* co-cultures utilizing pomegranate peels. The partially purified enzyme showed maximal relative activity at 37–65 °C for 10 min and kinetics of thermal inactivation energy at a high point at 60 °C for 0.040/min. The half-life was 37 °C for 58.6 min, temperature coefficient Q_10_ of tannase was maximal for 1.38 between 40 and 50 °C, and the activation energy was 17.42 kJ/mol. The enzyme activity peaked in the pH range of 4–8, and the maximum relative activity (100.6%) for tannase was achieved at pH 6. The K_m_ and V_max_ values for purified enzymes using tannic acid were 7.3 mg/mL and 3333.33 U/mL, respectively. The enzyme reduced the total tannin content in all tannin-rich substrates after 12h. The gallic acid (GA) had total phenols of 77.75 ppm and antioxidant activity of 82.91%. It was observed that the GA as antimicrobial influencer exhibited the largest inhibitory zone diameter (IZD) of 31 ± 1.0 mm against *Pseudomonas aeruginosa* ATCC27853. The GA minimum inhibitory concentration value was ranged from 7770.0–121.41 µg/mL. The obtained GA showed a bactericidal effect against all bacterial strains except *Shigella sonnei* DSM5570 and *Salmonella typhi* DSM17058, which showed bacteriostatic behavior.

## Introduction

Gallic acid (GA), one of the crucial industrial and therapeutic significant molecules with a demand of over 10,000 tons per year, can be produced by tannase in various ways. Tannins are present in angiosperms, gymnosperms, and pteridophytes and are primarily found in plant leaves, roots, bark, and fruit^1^. There are several uses for various plants' peel, leaves, oil, and juice, including pomegranate, guava, banana, grapes, and orange. Due to their abundance and rich content of antioxidants, including flavones, anthocyanidins, alkaloids, luteolin, tannins, and others^2–4^, they are also high in luteolin. Pomegranate peel wastes generate bioactive compounds from plants among the fruits described because they are particularly rich in polyphenolic compounds. This fruit has antioxidant effects and slows the oxidation of vitamin C due to its high tannin content, namely gallotannic acid, which produces gallic acid when hydrolyzed^5, 6^.

A significant amount of tannic acid is also frequently present in tannery effluents. The toxic tannery effluent's tannin load can be reduced with the help of tannase, allowing for the possibility of lowering operating costs. The accessibility of various agro-industrial residues paves the way for the most significant possible utilization of tannase production for tannin degradation and, ultimately the output of GA^7^. GA is a common secondary metabolite found in most plants (3,4,5 trihydroxy benzoic acid). A slightly colorless or slightly yellow crystalline solid, it is. Its chemical formula is C_7_H_6_O_5_, with a molecular weight of 170.11954 g/mol. Additionally, it has a 210 °C melting point and a 235 °C to 240 °C decomposition range, which results in the production of carbon dioxide and carbon monoxide, 1.69 kg/L (20 °C) is its density. It is insoluble in benzene, chloroform, ether petroleum, and alcohol but soluble in water, ethanol, glycerol, and ether^8^.

On a large scale, GA is created by tannase, a glycoprotein esterase breaking down tannic acid. Tannase from microbial sources has been suggested for the degradation of natural tannins over the past three decades^1^. Tannin bioconversion by microbial tannase can efficiently produce significant amounts of GA^6^.

GA and tannase production are linked because tannase catalyzes the depolymerization of hydrolyzable tannins, resulting in the release of GA. *Aspergillus* sp., *Rhizopus* sp*.*, and *Penicillium* sp. have been the most common filamentous fungi involved in tannin bioconversion, even individually or in co-cultures. Along with them, various other fungi from the genera *Trichoderma* sp., *Fusarium* sp., *Chaetomium* sp., *Rhizoctonia* sp., and others have been reported for their ability to degrade tannins, particularly hydrolyzable tannins. *Aspergillus* sp. has been the most potent and extensively studied tannase producer among the existing fungal sources^9^.

Fungal co-culture has important roles in increasing the activity of valuable enzymes such as cellulase, laccase, pectinase, tannase, etc., and improving the yield of essential products such as gallic, botulinic, and oleanolic acids. Also, It offers the unique advantages of low cost and simple operation and does not require expensive chemicals and complex gene-level manipulation^10^. *Aspergillus* sp. and *Penicillium* sp. fungi, in particular, primarily through fermentation^11^. In a solid medium (SSF) and submerged (SMF), Costa, et al.^12^ obtained tannase and GA from *Aspergillus tamarin*. Better results were obtained when *A. tamarin* and *A. japonicus* were combined to produce tannase and GA^13^. Also, tannase from co-culture of *Penicillium chrysogenum* + *Trichoderma viride* was used to hydrolyze tannic acid for the production of gallic acid (84 ± 2 U/g/min) using grape's peel as a substrate^14^.

This metabolite exhibits a range of bioactivities, including antioxidant, antimicrobial, anti-inflammatory, anti-ulcerogenic, and anticancer^15^. Recently, some studies have been published relating the effect of GA before the formation of amyloid plaques, considered the initial step in Alzheimer's disease^16^. In addition to medicinal aspects, GA is applied in other areas. Its first application was in the skin and leather industry as a chelating agent^12^. The first photographs used GA as a developer^17^. GA is used to synthesize trimethoprim, an antimicrobial agent, and as a preservative in food and beverages, primarily because of its power to kidnap free radicals^18^.

Additionally, the goal of the current study was to i) partially purify and characterize the tannase produced by combining the cultures of *A. niger* A8 + *T. viride* from pomegranate peels using the SSF technique. ii) utilize the gallic acid end product's antibacterial and antioxidant properties as well as its ability to remove tannin from agricultural residues.

## Materials and methods

### Pathogenic strains

Seven pathogenic bacterial strains were used to investigate the antibacterial activity, namely *Bacillus cereus* ATCC11778, *Escherichia coli* ATCC 8739, *Enterococcus faecalis* ATCC 7080, *Klebsiella pneumonia* ATCC00607, *Pseudomonas aeruginosa* ATCC27853, *Salmonella typhi* DSM 17,058, and *Shigella sonnei* DSM5570. These strains were taken from Microbiological Resource Centers (MIRCEN), Cairo, Egypt. Pathogenic bacterial strains were maintained on glucose agar^19^ and kept in the refrigerator at 4 °C.

### Tannase crude enzyme

Extracellular fungal tannase crude enzyme with 12.93 U/mg specific activity was obtained from the cultural consortium *Aspergillus niger* A8 + *Trichoderma viride* (5:5% v/v inoculum size) utilizing pomegranate peels with SSF technique through Ahmed and Abou-Taleb^20^.

### Partial purification of tannase by ammonium sulfate precipitation

Fractional precipitation with ammonium sulfate followed by dialysis partially purified crude tannase. According to Kumar, et al.^21^, different amounts of solid ammonium sulfate were separately added to 1L of a crude enzyme to attain 0–80% saturation at 4 °C and stirred. The crude enzyme was placed in an ice bath, and solid ammonium sulfate was gradually dissolved to achieve an initial 20% saturation at 4 °C. The mixture was centrifuged (SIGMA 2–16 P centrifuge, USA) at 4°C for 10 min at 10,000 rpm. The precipitated protein pellet was thrown away. More solid ammonium sulfate was added to the supernatant to reach 40% saturation at 4 °C. It was centrifuged after another night at 4 °C. More ammonium sulfate was added to the supernatant to achieve 60% saturation at 4 °C. It was centrifuged after another night at 4 °C. The precipitated protein pellet was then collected, and additional ammonium sulfate was added to the supernatant to achieve 80% saturation at 4 °C. The supernatant was discarded in the same manner as previously mentioned. The precipitate from each source was dissolved individually in a small amount of 0.1M phosphate buffer pH 7.0 and then dialyzed (Spectra/PorR, VWR 2003 dialysis membrane) overnight at 4 °C with twice changes of the buffer to remove ammonium sulfate, which inhibits the catalytic function of an enzyme. Total proteins and enzyme activity were measured in the method described below.

#### Tannase activity estimation

It was determined using a UV spectrophotometric method, as described by Ahmed and Abou-Taleb^20^. One milliliter of the crude enzyme (filtrate) was added to a 4 mL solution (consisting of 0.35 g tannic acid dissolved in 100 mL citrate buffer (0.05M and pH 5.5) and mixed well. The mixture reaction was incubated in a water bath (WB series standard model, 12 l, WB-12; Germany) at 37 °C, and 0.2 mL of the reacting compound was withdrawn at zero time (t0) and after 30 min of incubation time (t1). The enzyme reaction was stopped by adding ethanol 95% (2 mL v/v). The absorbance of the t0 and t1 was measured using a UV spectrophotometer (Chrom Tech CT-2200 UV/Vis) at 310 nm. The absorbance of the t_1_ and t_2_ were scored at 310 nm using a UV spectrophotometer (Chrom Tech CT-2200 UV/Vis). One unit (U) of tannase activity was considered the enzyme needed to hydrolyze 1μmol of ester per 1 min per mL. The enzyme activity was calculated according to the following Eq. (1):

1 $$\mathrm{Enzyme\, activity\, }(\mathrm{U}/\mathrm{mL}) = 114\mathrm{ \times }\left[(\mathrm{At}1-\mathrm{ At}2) / (\mathrm{t}2-\mathrm{t}1)\right],$$

where A is the absorbance and t is the time in minutes.

#### Gallic acid concentration (GAC)

GAC was determined using the method proposed by Balouiri, et al.^22^. After being diluted 100 times in acetate buffer (0.2 M, pH 5.0), the Gallic acid (GA) was measured in culture filtrate and recorded at 255 nm and 294 nm using a UV spectrophotometer (Chrom Tech CT-2200 UV/Vis). The following Eq. (2) was used to determine the GAC (mg/mL) using a specific extinction coefficient:

2 $$\mathrm{GAC }\left(\frac{\mathrm{mg}}{\mathrm{mL}}\right)= 21.77 \left(\mathrm{A}254.6\right)-17.17 (\mathrm{A}294.8),$$

where A is the absorbance.

#### Protein estimation

Protein concentration was assessed using the Bradford^23^ procedure and a bovine serum albumin standard.

### Partially purified tannase characterization

The optimum temperature for tannase activity and stability: The enzyme was characterized by the following of Wan, et al.^24^. The optimal temperature was found by assessing activity at optimum pH with varying temperature degrees (25, 37, 40, 45, 50, 55, 60, 70, 75, and 80 °C) for 10 min. The enzyme activity and kinetics of the activation energy (EEA) and thermal deactivation (K_d_) and the temperature coefficient (Q_10_) were calculated as mentioned below in Eqs. (3), (4), (5), (6), (7), (8), (9).

#### The activation energy (Ea)

It was estimated using the Arrhenius plot slope (− Ea/R)^25^. The activation energy is the minimum required energy for a chemical reaction.

3 $$\mathrm{Ln K}= (-\mathrm{Ea}/\mathrm{RT}) +\mathrm{ ln A}$$

4 $${\mathrm{E}}_{a} = -\mathrm{RT x ln }\left(\frac{\mathrm{K}}{\mathrm{A}}\right),$$

where: R = Gas constant. It is equal to 8.314 J/ (K/mol); T = Temperature of the surroundings, expressed in Kelvins; K = Reaction rate coefficient. It is measured in 1/sec and depends on temperature; A = Pre-exponential factor (also called the frequency factor) is expressed in 1/sec. This coefficient does not vary with temperature and is constant for a reaction.

The Arrhenius equation is rearranged as above it is a linear Eq. (5) with the form:

5 $${\text{y }} = {\text{ mx }} + {\text{ b}},$$

where: y is ln (K), m is − Ea/R, x is 1/T and b is ln A. − Ea/R is slope and ln A = intercept.

#### Thermal deactivation (K_d_)

The K_d_ constant at each temperature and the half-life (T1/2) were calculated using Eqs. (6), (7), (8)^25^:

6 $$\mathrm{ln A }=\mathrm{ln Ao }+\mathrm{ Kdx t}$$

7 $$\mathrm{Kd}= \mathrm{ln }(\mathrm{A}1/\mathrm{A}0)/\mathrm{t}1-\mathrm{t}0$$

8 $${\text{T1}}/{2} = {\text{ ln 2}}/{\text{K}}_{{\text{d}}} .$$

#### The temperature coefficient (Q_10_)

It represents the factor by which the rate (R) of a reaction increases for every 10-degree rise in the temperature (T). The Q_10_ measures the degree to which a biological process depends on temperature. Q10 was calculated using the following Eq. (9)^26^:

9 $$\mathrm{Q}10 ={{\left(\frac{R2}{R1}\right)}^{\left(\frac{10}{T2-T1}\right)}},$$

where: R1 is the measured reaction rate at temperature T1 (where T1 < T2); R2 is the estimated reaction rate at temperature T2 (where T2 > T1); T1 is the temperature at which the reaction rate R1 is measured (where T1 < T2); T2 is the temperature at which the reaction rate R2 is measured (where T2 > T1).

The optimum pH was determined using different buffers (0.1 M) with different pH values: glycine—HCl buffer (pH 1.5–2.0), sodium acetate buffer (pH 3.0–5.0), sodium phosphate buffer (pH 6.0–8.0), and borate buffer (pH 9.0–10.0) for 10 min at optimum temperature. To investigate pH stability, the enzyme solution was dissolved in 0.05 M buffer solutions (1:1), citrate buffer (pH 3.0–6.0), phosphate buffer (pH 7.0–8.0), and Tris–HCl (pH 9.0–10.0), and then incubated for 15, 30, 45, and 60 min.

The effect of substrate concentration on tannase activity (K_m_ and V_max_) was investigated. The partially purified enzyme was incubated with tannic acid at various concentrations (0, 0.175, 0.350, 0.525, 0.700, 0.875, 1.050, and 1.225 mg/mL) in the reaction mixture. Under standard assay conditions, each substrate concentration determined the activity per unit of time. The obtained enzyme kinetics data were plotted using the Michaelis–Menten, Lineweaver–Burk, and Hanse-Woolf plots to calculate K_m_ and V_ma_x values following Eqs. (10), (11), (12) according to Clarke^27^.

### Michaelis Menten equation

10 $$\mathrm{V }= \left(\frac{\mathrm{ Vmax }[\mathrm{S}] }{(\mathrm{Km }+ [\mathrm{S}]) }\right)$$

The Equation is nonlinear.

#### Lineweaver–Burk

Plot is generated from 1/V versus 1/ [S] data. The linear Equation used to determine the parameters:

11 $$1/\mathrm{V}= (\mathrm{Km}/\mathrm{Vmax}) (1/ [\mathrm{S}]) + 1/\mathrm{Vmax}.$$

The Equation of a straight-line y = mx + b.

The slope of the line = K_m_/V_max_ is the x, and the y-intercept is 1/V_max_.

#### Hanes Woolf plot

There is another equation to convert the Michaelis Menten equation to a straight line:

[S]/V is plotted against [S]. Here, the linear Equation is of the form:

12 $$\frac{\left[\mathrm{S}\right]}{\mathrm{V}}= \left(\frac{1}{\mathrm{Vmax}}\right)\left[\mathrm{S}\right]+\frac{\mathrm{Km}}{\mathrm{Vmax}},$$

where, the slope is 1/V_max_, and the intercept is K_m_/V_max_. V = Initial velocity of the reaction; K_m_ = Michaelis constant; V_max_ = Maximum initial velocity; [S] = Substrate concentration.

The percentage of relative tannase activity was calculated by comparing the activity of a treated enzyme to that of an untreated enzyme (control), which was assumed to be 100%.

## Applications of fungal GA (tannase end product)

### Tannin-rich wastes hydrolysis

The liberated GA at the desired intervals was used to estimate the potential use of tannase in tannin-rich waste hydrolysis. The dried form of each commercial contained three tannin-rich raw materials: powders of banana peel (BPS), guava leaves (GLS), and pomegranate peel (PPS) collected from Cairo, Egypt's local markets. Partially purified enzyme (0.5% v/v) was added to the waste (0.5 g), thoroughly mixed, and incubated at 55 °C for 24 h. GAC (mg/mL) released following enzymatic breakdown was measured using the method previously mentioned. The total tannin content of each material was estimated using a procedure outlined in Pinto, et al.^28^. In brief, 2.5 mL of Folin-Ciocalteu's phenol reagent and 0.5 mL of a tenfold diluted sample were combined, incubated for 5 min, and 2 mL of 20% (w/v) Na_2_CO_3_ was added. At room temperature, the mixture was incubated for 60 min. At 760 nm, the absorbance concerning the prepared blank was observed. The mg of tannin was calculated using a tannic acid standard. To calculate the percentage of tannin degradation hydrolysis, use Eq. (13) below. Equations (14) and (15) were used to calculate the conversion coefficient (%) and GA productivity^29^.

13 $$\mathrm{Tannin \,degradation\, \%}=\left(\frac{\mathrm{Tannin\, concentration \,before\, hydrolysis\, }-\mathrm{\, Tannin\, concentration\, after \,hydrolysis}}{\mathrm{Tannin \,concentration\, before\, hydrolysis }}\right)\mathrm{\times}100$$

14 $$\mathrm{Conversion \,coefficient\, }(\mathrm{\%})= \left(\frac{\mathrm{GAC\, }(\mathrm{mg}/\mathrm{mL})}{\mathrm{Tannin \,residual \,concentration} (\mathrm{mg}/\mathrm{mL})}\right)\mathrm{\times} 100$$

15 $$\mathrm{GA\, productivity\, }(\mathrm{mg}/\mathrm{mL}/\mathrm{h}) = \left(\frac{\mathrm{GAC \,at \,the\, end\, of\, fermentation\, }(\mathrm{mg}/\mathrm{mL}) }{\mathrm{Total\, fermentation \,time\, }(\mathrm{h})}\right)\mathrm{\times} 100$$

### Antioxidant activity and total phenol content

The free radical scavenging ability of fungal GA was measured using the method described by Marques et al.^30^ with some modifications. A mixture of 2 mL of 160-fold diluted fungal GA and 2 mL of 0.1 mmol/L 2,2-Diphenyl-1-picrylhydrazyl (DPPH) methanolic solution was prepared. After vigorously shaking the mixture and leaving it in the dark for 20 min, the absorbance at 517 nm was measured with a UV spectrophotometer (Chrom Tech CT-2200 UV/Vis). According to Folin-Ciocalteu's method, the total phenol content of fungal GA was determined^31^. The test sample was transferred to an Erlenmeyer flask (100 mL) in a GA amount of about 0.1 mL, and the final volume was raised by adding distilled water to reach 46 mL. After that, 1.0 mL of the Folin-Ciocalteu's reactive solution was added, and the mixture was left to sit for 3 min at room temperature. The previously mentioned solution was combined with 3 mL of sodium carbonate (2% w/v). After 30 min, the absorbance at 760 nm was then determined. The total phenol was expressed as GA equivalents based on the calibration curve.

### Antibacterial susceptibility of fungal GA using agar well diffusion assay

According to the technique described by Sen and Batra^32^, standard inoculums for pathogenic bacterial strains were produced. A loop of the active bacterial growth was used to make the bacterial fresh inoculant, which was then shaken at 150 rpm in flasks for 24 h at 37 °C until it reached the 0.5 MacFarland standard for turbidity. The tested bacteria had a standard inoculum of 1 × 10^5^ colony-forming units (CFU)/ mL. The agar well diffusion method was used to evaluate the antibacterial activity of the fungal GA against the tested pathogenic bacterial strains^22^. Wells of 8 mm in diameter were established on the inoculated plates with a sterile cork borer. Then 100 μL of standard inoculum of tested pathogen and 100 μL GAC (77.7 mg/mL) were added into the established wells. The plates remained standing until the samples were fully absorbed and incubated at 37° C for 24 h. The antibacterial activity was assayed by measuring the inhibition zone diameter (IZD) around the colonies. Ampicillin (Gr^−ve^ bacteria, 1000 μg/mL) and Streptomycin (Gr^+ve^ bacteria,1000 μg/mL) were positive controls. Activity index (AI) was calculated (Eq. (16)) according to Singariya et al.^33^.

16 $$\mathrm{AI}=\frac{\mathrm{The \;inhibition \;zone \;diamete}}{\mathrm{r \;standard \;antibiotic \;diameter}}$$

According to the Clinical and laboratory standard Institute^34^, a dilution method was used to determine fungal GA minimum inhibitory concentration (MIC). GA was serially diluted twice to achieve final concentrations of 7.77 mg/mL (control), 3.88, 1.94, 0.97, 0.485, 0.242, and 0.121 mg/mL by first adding 1 mL of stock to the first tube that contained 1 mL of distilled water and then adding 1 mL to each of the following tubes. As previously mentioned, these dilutions were put into the wells created in the inoculated plates.

The minimum bactericidal concentration (MBC) of fungal GA: the lowest GA concentration inhibiting the growth of pathogenic bacterial strains^35^. The MBC value was calculated by sub-culturing from the MIC-tested plates that did not grow. GA effect on pathogenic bacterial strains was calculated as the MBC/MIC ratio. When the MBC/MIC ratio was 2 or 4, the action was classified as bactericidal or bacteriostatic^36^.

### Statistical analysis

The obtained data was analyzed using IBM^®^ SPSS^®^ Statistics Server Version 23.0. (2015), as Muijs^37^ suggested at the 5% confidence level.

### Statement

With the permission of the local market, samples of tannin-rich raw plant materials were taken. The authors indicate that all actions were taken under the regulations and rules that were in effect.

## Results and discussion

### Partial purification of tannase by ammonium sulfate precipitation

Enzyme purification is typically unnecessary for commercial enzyme use, but high-purity enzymes are needed for pharmaceutical and clinical applications. Tannase from microbial sources has frequently been purified using conventional purification methods. These methods include separating the culture from the fermentation medium and selective concentration by precipitation using ammonium sulfate or organic solvents, according to Gupta et al.^38^.

The fungal crude enzyme was partially purified by ammonium sulfate precipitation at a range of concentrations (from 0–20%, 20–40%, and 40–60% w/v) to determine the optimum concentration for partial purification. According to Table 1, the specific activity gradually increased up to 14.78 U/mg protein at a saturation fraction of 40–60% with a purification fold of 1.14, removing some non-enzymatic proteins while recovering about 61.83% of the total tannase. Comparatively, saturation fractions of 0–20% and 20–40% led to tannase activities of 271.98 and 216.70 U/mL, protein concentrations of 20.45 and 15.94 mg/mL, and specific activities of 13.30 and 13.59 U/mg protein, respectively. Also, purification folds of 1.03 and 1.05 and enzyme recovery of 88.31 and 70.36% were obtained.

**Table 1:** Partial purification by ammonium fractional precipitation of tannase enzyme from *A. niger* A8 + *T. viride* utilizing pomegranate peels with SSF technique.

Ammonium sulphate saturation%	Protein content (mg/mL)	Tannase activity (U/mL)	Specific activity (U/mg protein)	Recovery (%)	Purification (-Fold)
Crude extract	23.81	308.00	12.93	100.00	1.00
0–20%	20.45	271.98	13.30	88.31	1.03
20–40%	15.94	216.70	13.59	70.36	1.05
40–60%	12.88	190.43	14.78	61.83	1.14

Nadaf and Ghosh^39^ reported that tannase of *Rhodococcus* NCIM 2891 had 0.295 U/mg specific activity after purification. In addition, Ma et al.^40^ found that *A. ficuum* Gim 3.6 tannase-specific activity after purification was 2.74-fold, with an enzyme activity recovery of 77.1%. In addition, Wan et al.^24^ mentioned that *A. niger* NL112 tannase specific activity of 57.96 U/mg proteins with a purification of 5.1-Fold. While tannase-specific activity was found to be 9.55 U/mg protein of the pure tannase from *A. awamori*^41^ and 10.22 U/mg protein of the pure tannase from *A. niger*^42^.

## Partially purified tannase characterization

### Optimum temperature and thermostability for tannase activity

**Figure 1:**
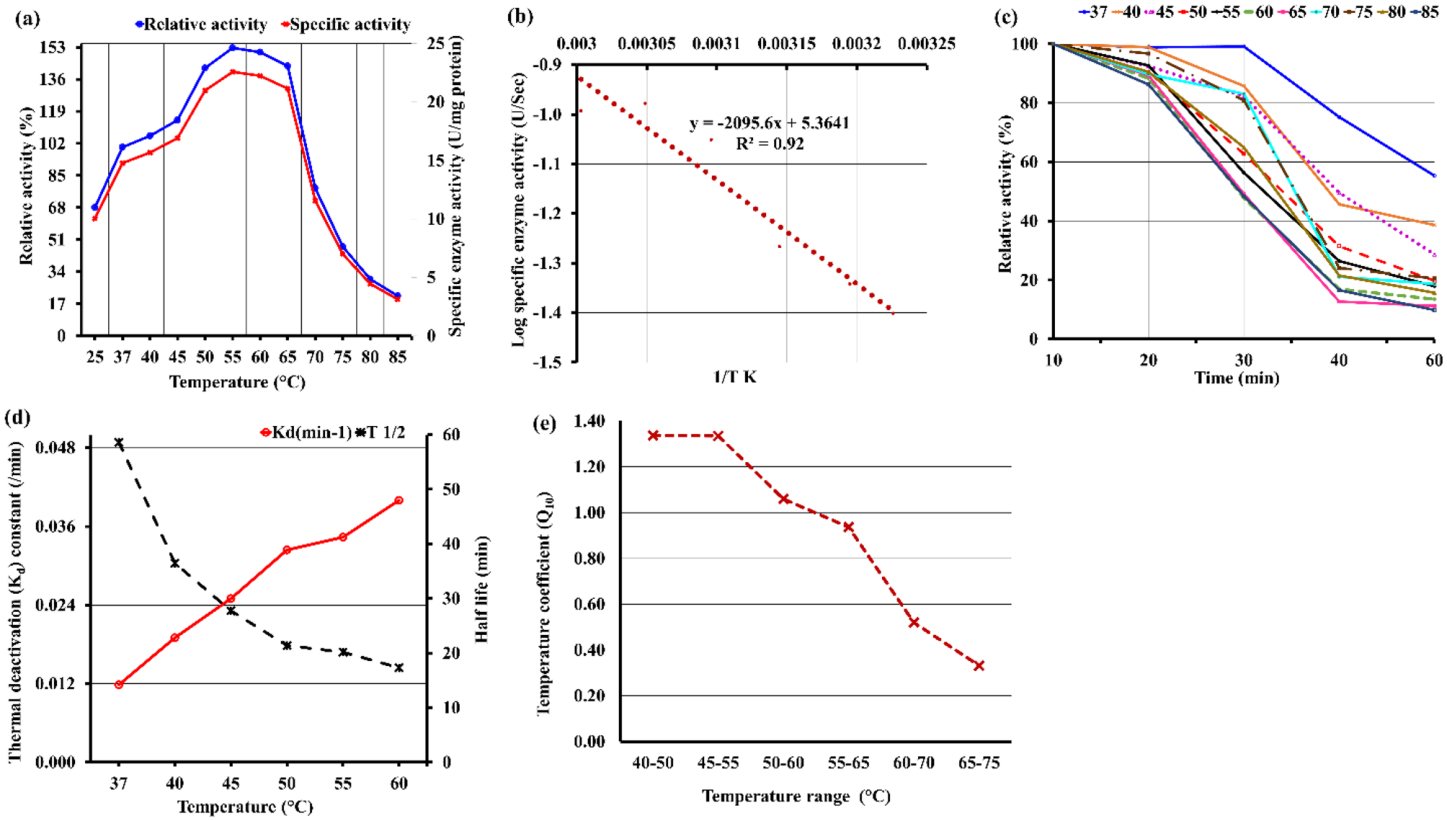
Temperature profile of partially purified tannase from *A. niger* A8 + *T. viride* utilizing pomegranate peels with SSF technique. (**a**) Optimum temperature, (**b**) The activation energy (Ea), (**c**) tannase thermostability, (**d**) Thermal deactivation energy K_d_ tannase and half-life (t_1/2_), (**e**) Temperature coefficient (Q_10_).

The activity was estimated by operating the assay for 10 min at various temperatures between 25 and 85 °C. The results in Fig. 1a demonstrate that as the temperature was raised to 65 °C, the relative enzymatic activity increased up to 152.7% (22.57 U/mg of specific activity), then gradually decreased to 143.0% (21.14 U/mg of specific activity), and finally reached 21.2% (3.14 U/mg of specific activity) at 80 °C. As mentioned by Wan, et al.^24^, The optimal temperature of tannase from *K. pneumoniae* MTCC 7162 and *A. niger* NL112 was found to be 40 °C and 45 °C, respectively.

The activation energy (Ea) is an important aspect from an industrial point of view, as it is relevant to know the Ea required for tannic acid hydrolysis by tannases for efficient reduction^25^. Data in Fig. 1b expressed the Arrhenius plot was applied to calculate the Ea. The Ea for fungal tannase was 17.42 kJ/mol. Furthermore, Ramos et al.^43^ revealed that the Ea for tannase produced by *Aspergillus niger* was 21.38 kJ/mol at 4.4 mM of methyl gallate. Moreover, Valera et al.^25^ reported that the optimal temperature for extracellular tannase II activity from *A. carbonarius* was 60 °C, and the Ea was 28.93 kJ/mol. Whereas de Sena, et al.^44^ found Ea for hydrolysis of tannic acid by tannase produced from *A. tamarii* URM7115 was 34.92 kJ/mol.

Regarding the enzyme exposed to temperatures ranging from 37 to 85 °C for 10–60 min to check its thermal stability. Results in Fig. 1c clearly show that fungal tannase activity was approximately stable at temperatures ranging from 37 to 85 °C for 10 min. The decrease in the thermal stability of tannase was first recorded after holding the enzyme at 45–85 °C for 20 min. On the other hand, the enzymes were denaturized by heating for 60 min exposed to test temperature levels ranging from 45 to 85 °C of lower than 20% or lower relative activity. The relative activity was maintained for 58, 39, and 34% when exposed to 37, 40, and 45 °C for 60 min. Yao, et al.^45^ revealed that tannases were stable at a temperature ranging from 30 °C to 60 °C. As well as, Valera et al.^25^ reported that *A. carbonarius* tannase II was stable at a temperature range of 20–60 °C for 120 min.

The thermal stability of tannase is characterized by three parameters: the thermal inactivation energy K_d_ at different temperatures, the half-life t_1/2_, and the thermal inactivation rate constant k. Tannase produced from *Aspergillus niger* A8 + *Trichoderma viride* thermal deactivation energy K_d_ where the native (active) form is transformed in the denaturated (inactive) form by a first-order unimolecular irreversible reaction. It is known that the inactivation of the enzyme is a first-order reaction^24^. The K_d_ was at a high point at 60 °C for 0.040/min, and the lowest was at 37 °C for 0.012/min, which means the K_d_ increased by 30% from 37 °C and 60 °C (Fig. 1d). Tannase half-life (t_1/2_) refers to the time needed to halve the activity of the enzyme. The half-life of tannase also followed the first-order kinetics (Fig. 1d). The half-life (t_1/2_) of the tannase was determined at various temperatures, and it was found to decrease rapidly and be maximum at 37 °C for 58.6 min while the lowest was scored at 60 °C for 17.3 min. Moreover, the tannase from *A. aculeatus* with a t_1/2_ of 17.5 min and k_d_ 0.039/min at 80 °C) and from *A. carbonarius* t_1/2_ of 62 min at 75 °C^46^. Whereas, the *A. tamarii* URM7115 tannase t_1/2_ of 247.55 min (4.13 h) at 40 °C and t_1/2_ of 106.54 min at 80 °C and the k_d_ increased from 0.13 to 0.39/min as the temperature increased from 40 to 80 °C^44^. While the t_1/2_ of tannase from *A. niger* NL112 decreased rapidly with increasing temperature and maintained the greatest t_1/2_ at 40 ~ 50 °C. It only remained 15% of the original activity after being incubated at 70 °C for 15 min^24^.

The effect of temperature on enzyme reaction is usually given in terms of the temperature coefficient Q_10_, which is the factor by which the velocity is measured on raising the temperature by 10 °C^47^. The temperature coefficient of enzyme reactions usually lies between 1 and 2. Results recorded in Fig. 1e clearly show that Q_10_ of tannase was maximal for 1.38 between 40 and 50 °C, whereas the Q_10_ value was at the lowest rank by 0.35 between 75 and 65 °C. According to de Sena, et al.^44^, the Q_10_ value of tannase from *A. tamarii* URM 7115 was 1.24. Whereas Jana, et al.^46^ calculated the Q_10_ value for tannase from *Bacillus subtilis* PAB 2 was 2.08. In another study by Cavalcanti, et al.^48^, the maximal tannase activity was achieved at 40–60 ºC for 120 min. Also, the soluble tannase produced by *A. fumigatus* CAS-21 presented an optimum activity at temperatures ranging from 30 to 40 °C for 60 min. As well as they showed a half-life (t_50_) of 60 min at 45 and 50 °C for 3 h^49^.

From the previous results, it can be concluded that 37–55 °C were suitable for tannase storage and stability for 10 min, it might be due to no auto-digestion of tannase occurring during the incubation period of 10 min. Temperatures above 55 °C promoted a reduction in enzymatic activity because the weak bonds in the three-dimensional structure of the protein break under high temperatures, causing its denaturation and loss of activity^48^.

### Optimum pH level and stability for tannase activity

**Figure 2:**
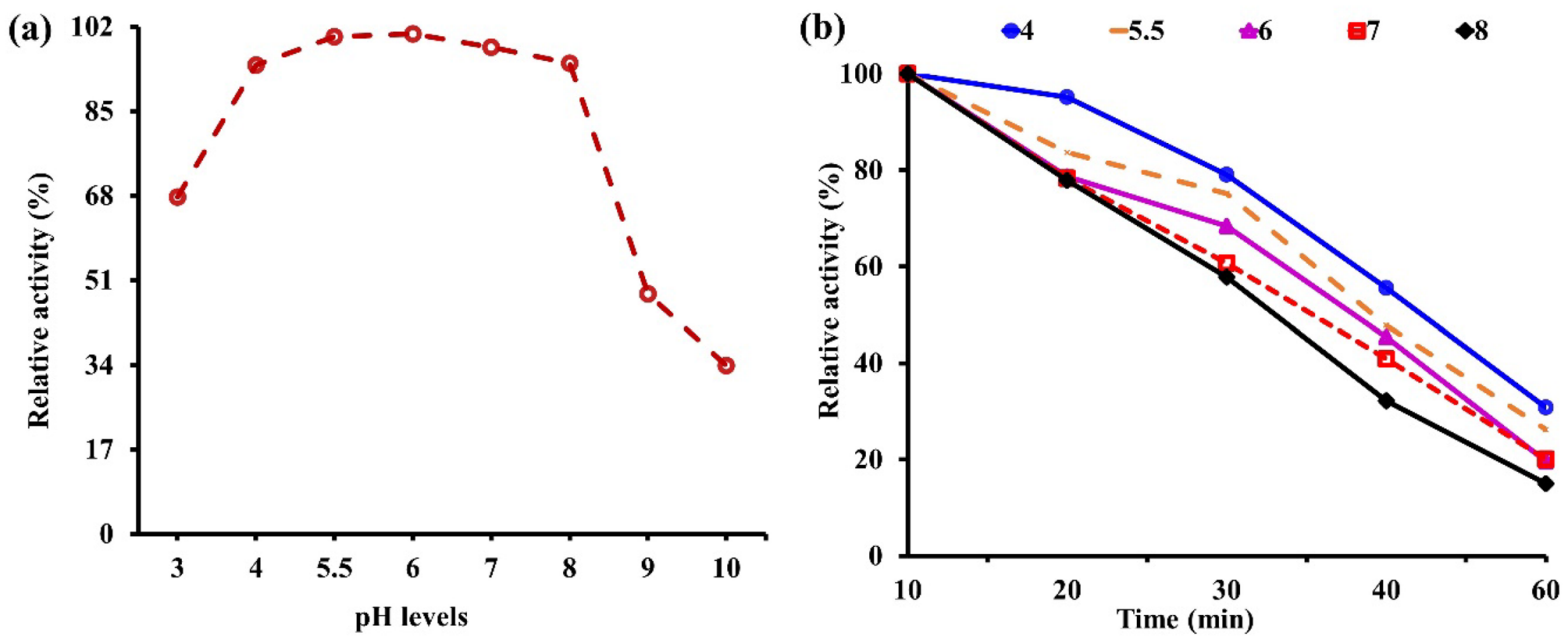
pH profile of partially purified tannase from *A. niger* A8 + *T. viride* utilizing pomegranate peels with SSF technique. (**a**) Optimum pH, (**b**) pH stability.

The tannase activity was found to have a wide pH range from 3 to 10. Results shown in Fig. 2a demonstrated that tannase activity peaked between pH values of 4 and 8, then decreased as pH levels increased or decreased to their best range of 5.5 to 6. The maximum relative activity (100.6%) for tannase was achieved at pH 6, followed by pH 5 (100% relative activity), while pH 9 and pH 10 scored the lowest relative with 48.22% and 33.8%, respectively. Like other protein denaturation, the rate of enzyme inactivation is typically highly influenced by the pH of the solution. According to Fig. 2b, which shows tannase stability, the relative activity of the enzyme decreased as pH increased over time, reaching its peak activity at pH 4.0–5.5 for 10 min. In contrast, when tannase was treated with all pH levels for 60 min, the relative activity was minimized by 65.0–80.0%.

Additionally, the results showed that tannase could maintain activity over 50% at pH level 5 for 40 min, reaching 55.6% of relative activity. Further, this enzyme was maintained at a high pH of 8 for 40 min with 57.8% relative activity (Fig. 2b). From the previous observations, it appeared that it tolerated acidic conditions better than alkali, which may be due to the abundance of acidic amino acids in its active sites, according to research by Sabu et al.^50^, the amino acid properties of the active site control the pH-dependent enzyme activity. While bacterial activity ranged from pH 4.5 to 5.5, most optimal pH values reported for fungal tannase activity ranged from pH 2.0 to 8.0^51^. As Wan et al.^24^ stated, the optimal *A. niger* NL112 tannase pH for the purified tannase active in an acidic environment was 5.0. Over the pH range of 4.0–6.0, it retained more than 80% relative activity. Whereas the maximal tannase activity was achieved at pH 5.0–6.0^48^.

### Effect of substrate concentration (V_max_ and K_m_)

**Figure 3:**
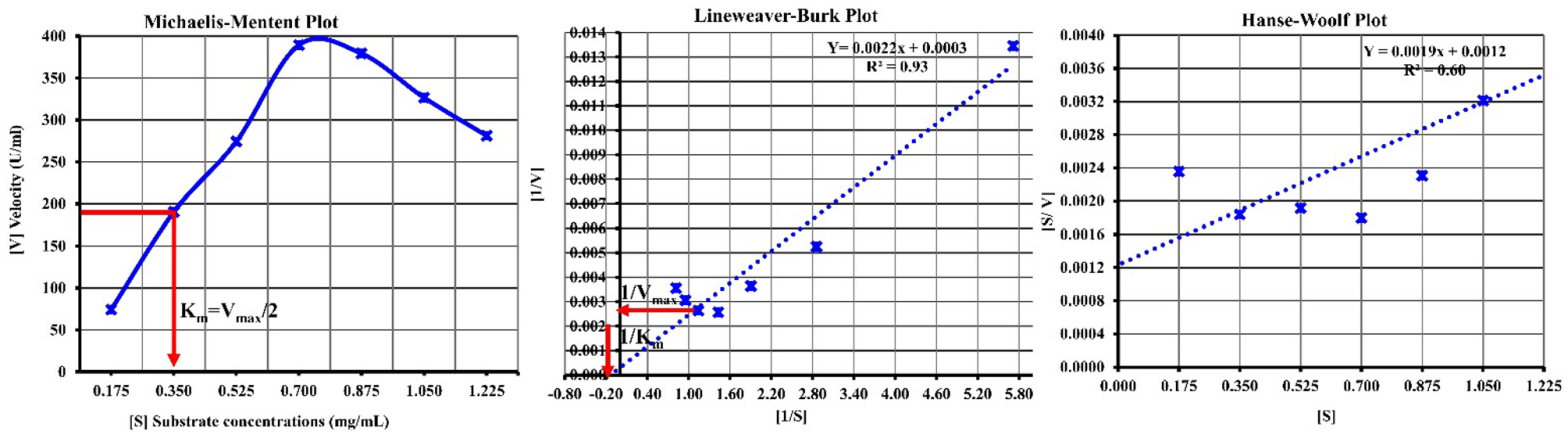
K_m_ and V_max_ values for partially purified tannase produced from *A. niger* A8 + *T. viride* utilizing pomegranate peels with SSF technique.

The enzyme source and particular substrate may define the values of kinetic constants of K_m_ and V_max_^27^. To simplify the evaluation of K_m_ and V_max_, the Michaelis–Menten equation, as 1/V = (K_m_/V_max_) X (1/[S]) + 1/V_max_. The enzyme activity was evaluated by varying its substrate concentration from 0.175 to 1.225 mg/mL. The results presented in Fig. 3 indicated a gradual increase in the activity by increasing the substrate concentration up to 0.70 mg/mL, and the enzyme recovery reached 389.61 U/mL with relative activity of 204.6%. Increasing the tannic acid concentration by more than 0.70% resulted in a decrease in the tannase activity. For the Lineweaver–Burk Plot, a straight line was obtained when 1/V was plotted as a function of 1/S as shown in Fig. 3, the vertical intercept is equal to 1/V_max_ being 0.0003, The slope represents K_m_/V_max_ being 0.0022 for partially purified tannase. The constant K_m_ for tannic acid was calculated to be 7.3 mg/ mL, while its maximum velocity, V_max_ was 3333.33 U/mL, as presented in Table 2. On the other hand, Hanse-Woolf Plot, as illustrated in Fig. 3, the vertical intercept equals 1/V_max_ being 0.0019, and the slope represents K_m_/V_max_ being 0.0012 for partially purified tannase. The constant K_m_ for tannic acid was calculated to be 0.63 mg/ mL, while its maximum velocity, V_max_, was 526.32 U/mL, as presented in Table 2. Moreover, Battestin and Macedo^52^ revealed that K_m_ and V_max_ values for *Paecilomyces variation* tannase were 0.61 µmol and 0.55 U/mL. Whereas Farias et al.^53^ reported that the K_m_ value was 7.49 mM for *Cryptomeria parasitic*us tannases using methyl gallate as substrate.

**Table 2:** Techniques for calculating K_m_ and V_max_ values for partially purified tannase produced from *A. niger* A8 + *T. viride* utilizing pomegranate peels with SSF technique.

Equation	Regression equation	K_m_/V_max_	1/V_max_	K_m_	V_max_
				(mg/mL)	(U/mL)
Lineweaver–Burk Plot	y = 0.0022x + 0.0003	0.0022	0.0003	7.3	3333.33
Hanse-Woolf Plot	y = 0.0019x + 0.0012	0.0012	0.0019	0.63	526.32

Tannase activity decreased when the tannic acid concentration was increased by more than 0.70%. Furthermore, data in Fig. 5 revealed that increasing substrate concentration rapidly increased reaction rate velocity (first-order reaction). While increasing substrate concentration, no further change in velocity was observed (zero order kinetic). This is because the enzyme-catalyzed reaction at different substrate concentrations is diphasic. The active site of the enzyme was not saturated at low concentrations, and as the number of substrate molecules increases, the active sites become available, and the enzyme operates at full capacity. In that case, the rate is unaffected by the concentration of the substrate^54^.

In the same line Wan et al.^24^, *A. niger* NL112 tannase K_m_ increased with temperature. It could be due to the changes in the molecular space structure of the tannase protein and the loss of some activity caused by excessively increased substrate^41, 43^.

## Applications of crude and partially purified Tannase

### Degradation of tannin-rich wastes

**Figure 4:**
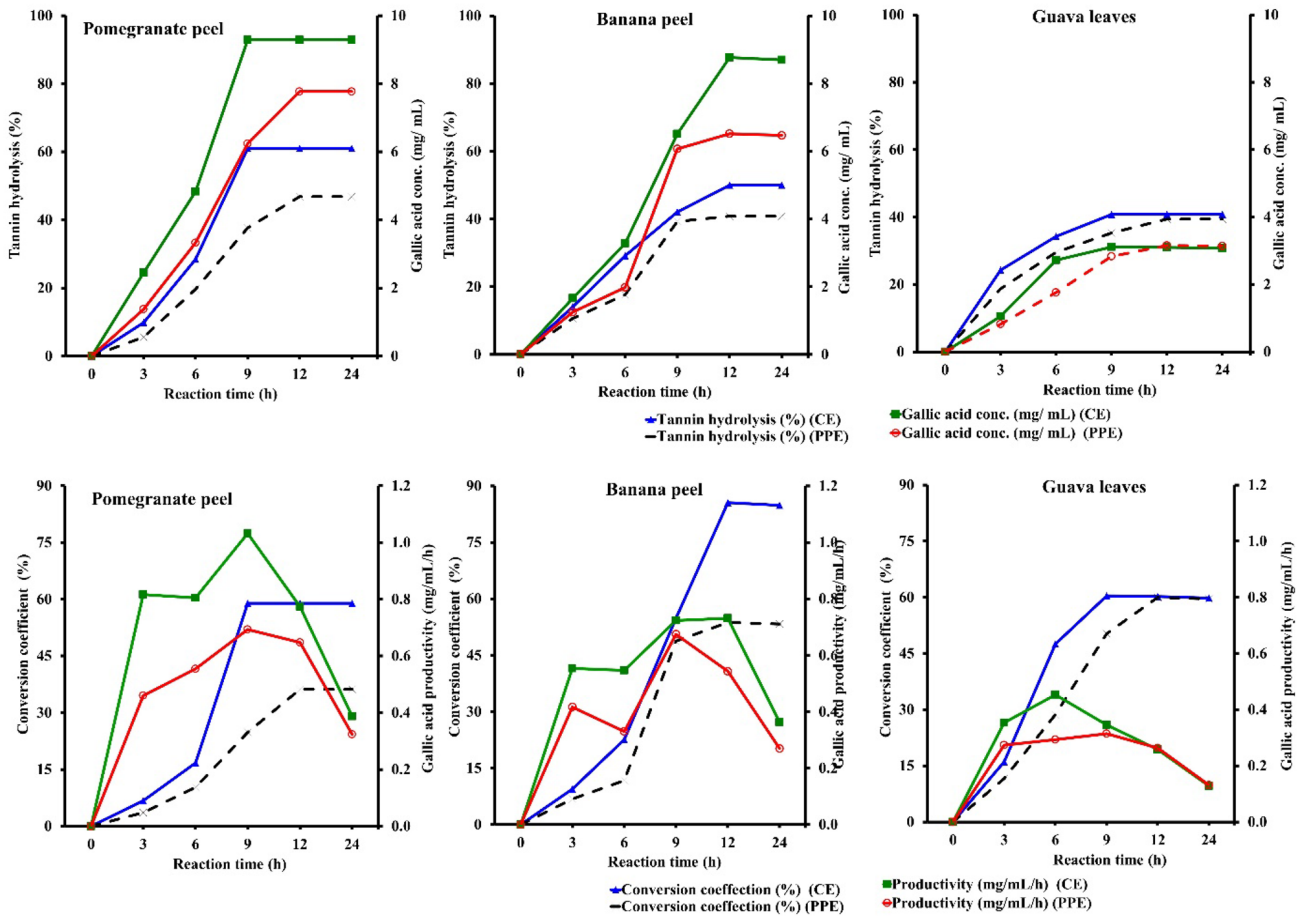
Tannin hydrolysis, conversion coefficient, gallic acid formation, and gallic productivity during degradation of tannin-rich wastes by crude enzyme and partially purified tannase enzyme produced from *A. niger* A8 + *T. viride.*

Conversion of tannin-rich wastes of PPS, BPS, and GLS into GA by *A. niger* A8 + *T. viride* crude enzyme and partially purified enzyme of tannase. As shown in Fig. 4, all treatments exhibited tannin hydrolysis to a varied extent. Tannins hydrolysis and GAC were gradually increased for crude and partially purified enzymes and reached a maximum peak after 24 h for PPS, BPS, and GLS. For crude enzymes, it was found that the percentage of tannin hydrolysis was 60.0, 50.0, and 40.0%, and GAC was 9.29, 8.77, and 3.11 mg/mL after 9, 12, and 9 h for PPS, BPS, and GLS, respectively. Thus, the efficiency of tannin-rich waste degradation increased by 20% and 10% in the case of PPS and BPS compared to GLS. It was found that the formation efficiency increased using PPS with 1.5-Fold and 1.2-Fold compared with BPS and GLS, respectively.

Moreover, the maximum conversion coefficient of wastes was 58.91, 85.56, and 60.39%, with GA productivity of 1.03, 0.73, and 0.35 mg/mL/h after 9, 12, and 9 h for PPS, BPS, and GLS, respectively. Whereas with partially purified enzyme treatment, it was found that the tannin hydrolysis was 46.9, 40.8, and 39.4%, and GAC was 7.77, 6.52, and 3.16 mg/mL for PPS, BPS, and GLS after 12 h, respectively. For partially purified enzyme treatment, the efficiency of tannin content hydrolysis increased by 6.1% and 7.5% in the case of PPS and BPS compared to GLS. For the GAC, it was found that the formation efficiency increased using PPS with 1.2-folds and 2.5-folds compared with BPS and GLS. In addition, the maximum conversion coefficient of wastes was 36.19, 53.75, and 59.96%, with productivity of 0.65, 0.54, and 0.26 mg/mL/h after 12 h for PPS, BPS, and GLS, respectively.

In comparison with crude enzyme and partially purified enzyme treatment for PPS, it was found that the tannin hydrolysis decreased by 14.1%, and for gallic concentration, it was reduced by 17% (− 1.19-fold reduction). When comparing the crude enzyme and partially purified enzyme treatments for BPS, the loss for tannin hydrolysis and GAC were 9.2% and 26% (− 1.43-fold reduction). On the other hand, the GLS was the lowest affected in tannin hydrolysis and gallic concentration compared with the other peels (PPS and BPE), as the reduction only scored 1.4% and 1.6% (− 1.01-Fold reduction). So, after all, the PPS was selected as the source for GA formation in the antimicrobial investigation. In a similar vein, many researchers have used tannase to reduce tannin content in various applications, such as pomegranate juice, where it was reported that 40% of the tannin had been removed^55^. *A. niger* tannase removed 73.6% of the tannin from fresh amla (*Phyllanthus emblica*) juice after 3 h of processing^56^. Tannase from *Penicillium atramentosum* has been shown to reduce the tannin content of tea extract by 74%, grape wine by 43.5%, and jamum wine by 38% after 3 h^57^. In a related study, after 3 h of treatment with *T. harzianum* crude tannase, the maximum tannin reduction was attained, and it was around 57%^58^. When *A. niger* MTCC 2425 was used to declare pomegranate, it was discovered that after 2 h with partially purified tannase (173 U/mg), tannin content had decreased by 56%^59^. Additionally, after being incubated for 2 h with crude tannase from *T. harzianum*, the tannin degradation efficiency of various agro-residues did not increase the production of GA^58^. Additionally, they showed that some tannin wastes and sources, such as amla fruits, jamun leaves, tamarind seeds, keekar leaves, and mulberry leaves, proved to be better substrates for tannase than tannic acid. The enzyme also demonstrated notable activity of 97, 74, 55, 66, and 88%, as well as tannin content of 1.22, 0.74, 0.66, 0.22, and 0.6 mg/mL, respectively, with pomegranate peels, guava leaves, guava park, eucalyptus bark, and mango leaves.

### GA (tannase end product) antioxidant and total phenolic content

**Figure 5:**
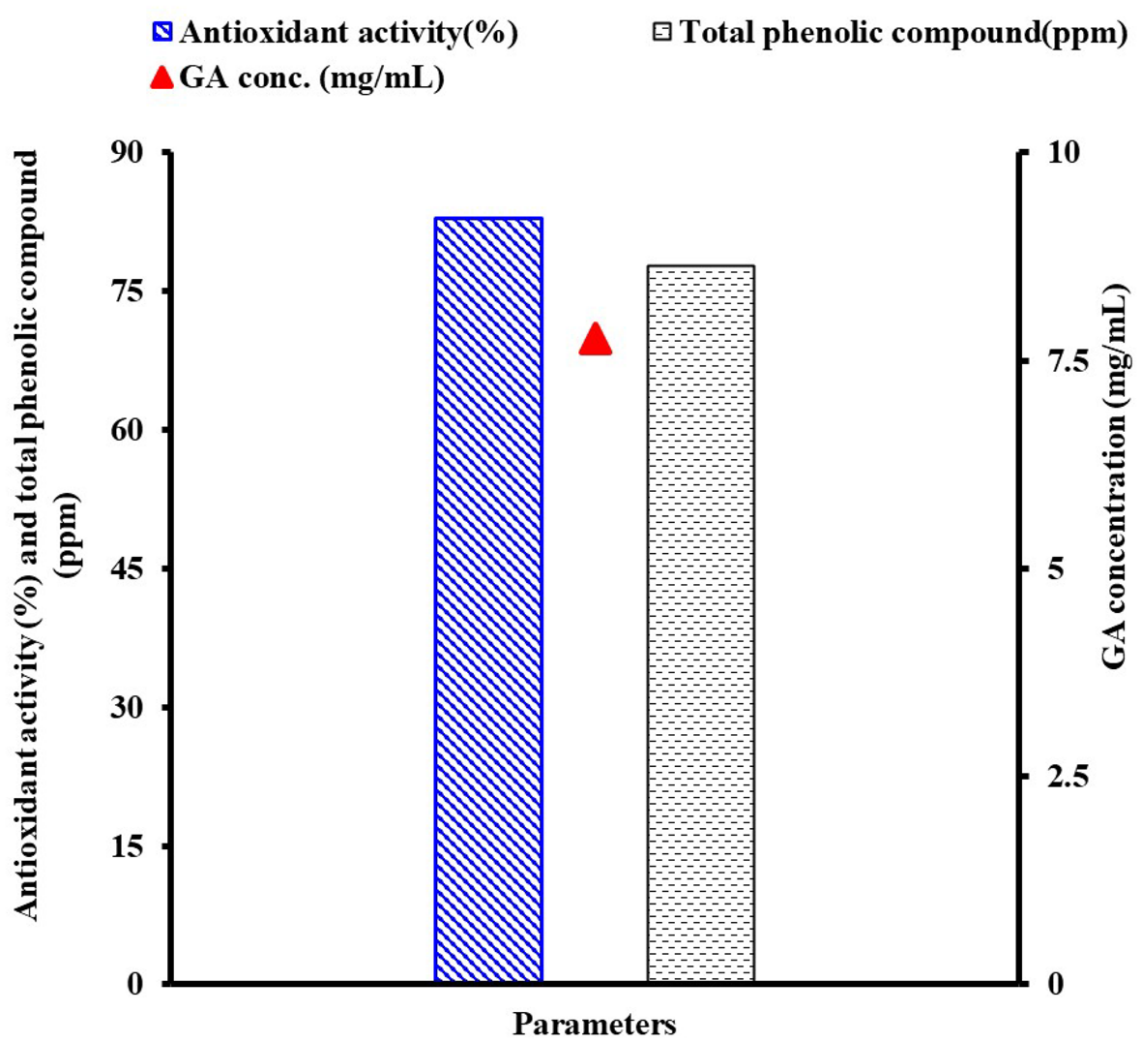
Antioxidant and total phenolic content of gallic acid.

 As shown in Fig. 5, GA produced (7.77 mg/mL) using PPS had a total phenolic compound score of 77.75 ppm, while its antioxidant activity increased considerably by 82.91%. Compared to sources without the enzyme treatment, treating sources containing tannin with tannase enzyme significantly increased GA, total phenols, and antioxidant activity^60, 61^. Without tannase enzyme treatment, the antioxidant activity of *P. charlesii* crude tannase for the tea leaves was 35%^62^. The Red grape (RGW) had the highest total phenols and antioxidants by 36.5 mg/L (ppm) and it was 166.5% larger than the White grape (WGW) and Moscato grapes (MGW) in the crude without tannase *Paecilomyces variotii* treatment of various grape wastes^63^. Additionally, it was found that tannase was most effective in the hydrolysis of polymeric polyphenolics as it increased total phenol content by 61.1% in RGW, 21.5% in WGW, and 33.2% in MGW compared to the corresponding untreated grapes water. The antioxidant activity of tannase from *A. nidulans* FT10 was 60%, in contrast^64^. Tannase was also added to green tea leaves, which resulted in a 27% increase in the antioxidant content^60^.

Furthermore, after exposure to the unprocessed enzyme tannase, black tea's antioxidant activity increased by 52.31% from its baseline activity^61^. Additionally, it was noted that the total phenolic content had increased to 12.48 mg GAE/g (dry weight). In a different study, the Saccharomyces cerevisiae CCMB 520 tannase was used to treat pitanga (*Eugenia uniflora*) and reach tannins juice. The total phenolic content was found to be 4855 mg/L, and the antioxidant activity was 69.41%^65^.

### Antibacterial activity evaluation of GA using agar well diffusion assay

**Figure 6:**
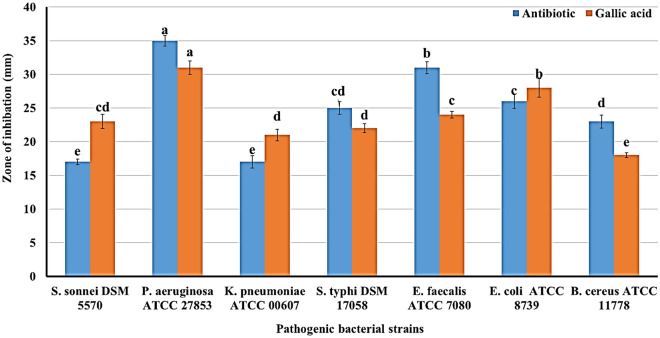
Inhibition zone diameter (mm) of antimicrobial effect of gallic acid against bacterial pathogenic strains, compared to the control antibiotics. Results are averages of 5 replicates. ^a,b^Values in the above column followed by the same superscript letter do not significantly differ from each other at *p* ≤ 0.05. Bar, standard division.

The antibacterial activity of the tannase enzyme end product (GA) from PPS with a concentration of 7.77 mg/mL was detected by the agar well-diffusion method, as recommended by Balouiri et al.^22^. The pathogenic bacterial strains were *B. cereus* ATCC11788 and *E. faecalis* ATCC7080 (as G^+ve^ bacteria) and *E. coli* ATCC8739, *K. pneumonia* ATCC00607, *P. aeruginosa* ATCC27853, *S. typhi* DSM17058 and *S. sonnei* DSM5570 (as G^-ve^ bacteria). Results in Fig. 6 showed that the produced GA exhibited high antimicrobial activity against pathogenic, especially G^-ve^ bacteria, compared with recommended antibiotics, namely streptomycin for G^+ve^ and ampicillin for G^−ve^, with a concentration of 1000 µg/mL. GA (at 7770.0 μg/mL) achieved an inhibition zone diameter (IZD) ranging from 18 ± 0.37 mm to 31 ± 1.00 mm. In comparison, antibiotics gave IZD ranging from 17 ± 0.40 mm to 35 ± 077 mm. The largest significant IZD (31 ± 1.00 mm) was recorded for *P. aeruginosa* ATCC27853 followed by 28 ± 1.36, 24 ± 0.51, 23 ± 1.05, 22 ± 0.66 and 21 ± 0.86 mm IZD for *E. coli* ATCC8739, *E. faecalis* ATCC7080, *S. sonnei* DSM5570, and *K. pneumonia* ATCC00607, respectively. In contrast, the lowest IZD was 18 ± 0.37 mm for *B. cereus* ATCC11788*.* The highest activity index (AI) was recorded *for S. sonnei* DSM5570, *K. pneumonia* ATCC00607*,* and *E. coli* ATCC8739 at 1.35, 1.24, and 1.08, respectively. The lowest activity was 0.77 and 0.78 for *E. faecalis* ATCC7080 and *B. cereus* ATCC11788, respectively. Various foodborne pathogens and bacteria, including *P. aeruginosa, Helicobacter pylori*, *E. coli*, *S. aureus*, *S. mutans*, and *L. monocytogenes* are susceptible to GA's antibacterial properties^66^. The antimicrobial effectiveness of tannase enzyme against *Streptococcus agalactiae, S. aureus*, *P. aeruginosa*, *S. flexneri*, and *K. pneumonia* increased with lower MIC and increased diameter of inhibition for all tested bacteria when combined with other antibiotics^64, 67^. Additionally, they stated that the proper antibacterial mechanism for the tannase enzyme and its GA end product is linked to the prevention of bacterial surface attachment, the disruption of quorum sensing, decreased cell viability, the destruction of cell membranes, and changes in the morphology of the bacteria. With 500, 250, 125, 62.5, 31.25, 15.63, 7.18, and 3.91 µg GA /mL, GA inhibited *Mannheimia haemolytica,* bovine respiratory disease associated-pathogens by 84, 78, 43, 38, 20, 20 and 3%, while *Pasteurella multocida* inhibited by 90, 79, 55, 40, 30, 20 and 6%, respectively^68^. Using the agar disc diffusion technique, the antibacterial activity of the GA from co-cultivated *Bacillus gottheilii* M2S2 and *Bacillus cereus* M1GT against food-borne pathogenic *E. coli*, *S. aureus*, and *Serratia marcescens* and showed a zone of inhibition of 20.0 , 16.0, and 13.0 mm, respectively^69^. It was thought that GA caused irreversible changes in membrane properties (charge, intra and extracellular permeability, and physicochemical properties) by changing hydrophobicity, decreasing negative surface charge, and causing local rupture or pore formation in cell membranes, resulting in leakage of essential intracellular^70,71^. TGA inhibited efflux pumps, which are a key mechanism in the development of AMR in *S. aureus* strains and *Pseudomonas* sp. and multidrug resistant *E. coli* strains^72^. It further emerged that GA antimicrobial activities were associated with various pathways within the cytoplasmatic membrane through destabilization, permeabilization, and inhibitory enzyme by oxidized products, possibly through reaction with sulfhydryl groups or more nonspecific interactions with proteins and inhibition of nucleic acid synthesis for both Gram-negative and Gram-positive bacteria^73^. Previously, the study reported that Gram-positive bacteria were more resistant than Gram-negative bacteria due to the addition of hydroxyl groups, followed by the substitution of hydroxyl groups to methoxy groups, resulting in increased GA activity. GA's antibacterial efficacy is associated with a mechanism that alters bacterial hydrophobicity, which is facilitated by its physicochemical surface properties. Gallic acid caused changes in the polar, nonpolar, and electron acceptor (c +) components of bacterial cells. It resulted in differential ability for increased electron acceptor, as seen in Gram-positive bacteria, and decreased electron acceptor, as seen in Gram-negative bacteria^74^. It was also electrophilic and had a strong reliance on bacterial surface components^68^.

### MIC of GA

Results in Table 3 demonstrated that GA MIC values ranged from 7770.0 to 121.41 µg/ mL against the pathogenic bacterial strains. The MIC value for *P. aeruginosa* ATCC27853 was 1942.5 µg/ mL, while it was 971.25µg/ mL for *B. cereus* ATCC11788*,* and the rest bacterial strains scored MIC for 242.81 µg/ mL with *E. coli* ATCC8739, *E. faecalis* ATCC7080, *S. sonnei* DSM5570, *S. typhi* DSM17058, and *K. pneumonia* ATCC00607. Results also show that the antibacterial potential at the tested concentrations that ranged from 3885.0 to 1942.5 µg/ mL had 100% of spectrum activity, which inhibited 7 tested strains, whereas, at concentrations of 971.25, 485.63, and 242.81 µg/mL, the GA recorded 85.7, 71.4, and 71.4% of spectrum activity which inhibited 6, 5, and 5 strains out of 7 tested pathogenic strains, respectively.

**Table 3:** Gallic acid MIC, MBC, and MIC/MBC ratio effect on pathogenic bacterial strains after incubation at 37 °C for 24 h.

Parameters	Pathogenic bacterial strains								
Gallic acid concs. (µg/mL)	*S. sonnei* DSM 5570	*P. aeruginosa* ATCC27853	*K. pneumoniae* ATCC00607	*S. typhi* DSM17058	*E. faecalis* ATCC7080	*E. coli* ATCC8739	*B. cereus* ATCC11778	Spectrum Activity (%)	
7770.0 (Control)	−	−	−	−	−	−	−	7/7	100
3885.0	−	−	−	−	−	−	−	7/7	100
1942.5	−	−	−	−	−	−	−	7/7	100
971.25	−	+	−	−	−	−	−	6/7	85.7
485.63	−	+	−	−	−	−	+	5/7	71.4
242.81	−	+	−	−	−	−	+	5/7	71.4
121.41	+	+	+	+	+	+	+	0/7	0
MIC value (µg/mL)	49.38	395	49.38	49.38	49.38	49.38	197.5		
MBC (µg/mL)									
7770.0 (Control)	−	−	−	−	−	−	−	7/7	100
3885.0	−	−	−	−	−	−	−	7/7	100
1942.5	−	+	−	−	−	−	+	5/7	71.4
971.25	+	+	−	+	−	−	+	4/7	57.1
485.63	+	+	−	+	−	+	+	3/7	42.8
242.81	+	+	+	+	+	+	+	0/7	0
MBC value (µg/mL)	197.5	395	49.38	197.5	49.38	98.75	395		
MBC/MIC Ratio	3.99	1	1	3.99	1	1.99	2		
Effect	Bacteriostatic	Bactericidal	Bactericidal	Bacteriostatic	Bactericidal	Bactericidal	Bactericidal		

− no growth, + growth.

Results are averages of 5 replicates, Bactericidal ≤ 2, and Bacteriostatic effect =  ≥ 2.

GA exhibited antimicrobial activity against the bacteria tested, with MIC values of 100, 1100, and 1250 µg/mL for *L. monocytogenes*, *E. coli*, and *P. aeruginosa*, respectively^70^. In contrast, *K. pneumoniae* has a higher MIC (9.75 μg/mL) than *S*. epidermidis (9.8 μg/mL) and *S. aureus* (19.5 μg/mL)^73^. The MIC for *S. flexneri* was at 2 mg/mL^67^. GA was active against *M. haemolytica* and *P. multocida* with MIC values of 250 and 500 μg/mL, respectively^68^. In contrast, the GA from the *Altingia excelsa Noronha* leaf extract had an inhibitory effect on *E. faecalis* with a MIC value of 12.25 µg/mL^74^.

### MBC of GA

Using the GA, the maximum obtained MBC value for *P. aeruginosa* ATCC27853 and *B. cereus* ATCC11788 was 971.25 µg/ mL. In comparison, it was 485.63 µg/mL for both *S. typhi* DSM17058 and *S. sonnei* DSM5570 and 242.81 µg/ mL for *E. coli* ATCC8739. At the same time, the MBC score for the rest of the bacterial strains scored MBC for 121.41 µg/ mL with *E. faecalis* ATCC7080 and *K. pneumonia* ATCC00607 (Table 3). In addition, the results show that the antibacterial potency of GA at the tested concentrations that range from 1942.5 to 3885.0 µg/ mL was 100% (7/7 strains), while at 971.25 µg/ mL, the activity was 71.4% (5 strains of a total 7 strains) and 57.1% (4 strains of a total 7 strains) for 485.63 µg/ mL. The GAC for 242.81 µg/mL displayed 42.8% spectrum activity against 3 strains among 7 of the tested pathogenic strains. Furthermore, GA had MBC of 5000 and 5500 g/mL for both Gr^+ve^ bacteria and 5250 and 500 g/mL for Gr^−ve^ bacteria *E. coli* and *P. aeruginosa*^70^. GA MBC value against *S. flexneri* was 8 mg/mL^67^.

The observed action of GA against the pathogenic bacterial strains is presented in Table 3. Results indicated that the GA had a bactericidal effect against all the tested bacterial strains with MBC/MIC ≤ 2 except *S. sonnei* DSM5570 *and S. typhi* DSM17058*,* which had a bacteriostatic impact with MBC\MIC ≥ 2.

## Conclusions

The specific activity of partially purified tannase from co-cultures of *A. niger* A8 + *T. viride* gradually increased to 14.78 U/mg protein at a 40–60% saturation fraction with a purification fold of 1.14. The enzyme's thermal and pH stability parameters were 37–65 °C and 4–6 for 10 min, and its thermal kinetic parameters were K_d_, t_1/2_, Q10, and Ea. They were 60 °C for 0.040/min, 37 °C for 58.6 min, 1.38 between 40 and 50 °C, and 17.42 kJ/mol, respectively. The K_m_ and V_max_ values were 7.3 mg/mL and 3333.33 U/mL, respectively. This enzyme was used to reduce tannins in three agricultural wastes, including PPS, BPS, and GLS, with hydrolysis rates ranging from 40.8 to 61.0% for the crude enzyme and from 39.4 to 46.9% for the partially purified enzyme. The GA end product was an antioxidant and antibacterial agent against foodborne pathogens. As a result, this enzyme and end product will be produced in a bioreactor and used on a large scale in the food industry.

## Data Availability

The authors declare that the article contains all the data established and analyzed during this investigation.

## References

[1] Lekshmi R, Arif Nisha S, Thirumalai Vasan P, Kaleeswaran B (2021). A comprehensive review on tannase: Microbes associated production of tannase exploiting tannin rich agro-industrial wastes with special reference to its potential environmental and industrial applications. Environ. Res..

[2] Yoshime LT, Melo ILPD, Sattler JAG, Torres RP, Mancini-Filho J (2019). Bioactive compounds and the antioxidant capacities of seed oils from pomegranate (*Punica*
*granatum* L.) and bitter gourd (*Momordica*
*charantia* L.). Food Sci. Technol..

[3] Ambigaipalan P, de Camargo AC, Shahidi F (2016). Phenolic compounds of pomegranate byproducts (outer skin, mesocarp, divider membrane) and their antioxidant activities. J. Agric. Food Chem..

[4] Kang SJ (2015). Inhibitory effects of pomegranate concentrated solution on the activities of hyaluronidase, tyrosinase, and metalloproteinase. J. Cosmet. Sci..

[5] Sundaralingam R, Niren AS, Premina S (2021). Optimization of pomegranate peel extracts for the bioconversion of the ellagitannins to ellagic acid using *Aspergillus niger*, *Rhizopus oryzae*, mixed culture. Int. J. Pharm. Pharm. Sci..

[6] Lekshmi R, Arif Nisha S, Kaleeswaran B, Alfarhan AH (2020). Pomegranate peel is a low-cost substrate for the production of tannase by *Bacillus velezensis* TA3 under solid state fermentation. J. King Saud Univ. Sci..

[7] Fraga-Corral M (2021). By-products of agri-food industry as tannin-rich sources: A review of tannins’ biological activities and their potential for valorization. Foods.

[8] Qu Y, Wang L, Mao Y (2022). Gallic acid attenuates cerebral ischemia/re-perfusion-induced blood–brain barrier injury by modifying polarization of microglia. J. Immunotoxicol..

[9] Dhiman S, Mukherjee G, Singh AK (2018). Recent trends and advancements in microbial tannase-catalyzed biotransformation of tannins: A review. Int. Microbiol..

[10] Xu S (2023). The Potential use of fungal co-culture strategy for discovery of new secondary metabolites. Microorganisms.

[11] Macedo GA, Matsuda LK, Battestin V (2005). Seleção de fungos produtores de tanase em resíduos vegetais ricos em taninos. Cienc. e Agrotec..

[12] Costa AD (2013). Production of tannase and gallic acid by *Aspergillus tamarii* in submerged and solid state cultures. Afr. J. Biochem. Res..

[13] de Melo AG (2013). Screening and identification of tannase-producing fungi isolated from Brazilian caves. Afr. J. Biochem. Res..

[14] Paranthaman R, Vidyalakshmi R, Murugesh S, Singaravadivel K (2009). Effects of fungal co-culture for the biosynthesis of tannase and gallic acid from grape wastes under solid state fermentation. Global J. Biotechnol. Biochem..

[15] Aithal M, Belur PD (2013). Enhancement of propyl gallate yield in nonaqueous medium using novel cell-associated tannase of *Bacillus massiliensis*. Prep. Biochem. Biotechnol..

[16] Jayamani J, Shanmugam G (2014). Gallic acid, one of the components in many plant tissues, is a potential inhibitor for insulin amyloid fibril formation. Eur. J. Med. Chem..

[17] Beniwal V, Kumar A, Sharma J, Chhokar V (2013). Recent advances in industrial application of tannases: A review. Recent Pat. Biotechnol..

[18] Bajpai B, Patil S (2008). A new approach to microbial production of gallic acid. Braz. J. Microbiol..

[19] Atlas RM, Synder JW (2011). Reagents, stains, and media: Bacteriology. Manual Clin. Microbiol..

[20] Ahmed AI, Abou-Taleb K (2019). Implementation of different fermentation techniques for induction of tannase and gallic acid using agro-residues substrates. Egypt. J. Med. Microbiol..

[21] Kumar CG, Joo H-S, Koo Y-M, Paik SR, Chang C-S (2004). Thermostable alkaline protease from a novel marine haloalkalophilic *Bacillus clausii* isolate. World J. Microbiol. Biotechnol..

[22] Balouiri M, Sadiki M, Ibnsouda SK (2016). Methods for in vitro evaluating antimicrobial activity: A review. J. Pharm. Anal..

[23] Bradford MM (1976). A rapid and sensitive method for the quantitation of microgram quantities of protein utilizing the principle of protein-dye binding. Anal. Biochem..

[24] Wan Y (2021). The change mechanism of structural characterization and thermodynamic properties of tannase from Aspergillus niger NL112 under high temperature. Appl. Biochem. Biotechnol..

[25] Valera LS, Jorge JA, Guimarães LHS (2015). Characterization of a multi-tolerant tannin acyl hydrolase II from *Aspergillus carbonarius* produced under solid-state fermentation. Electron. J. Biotechnol..

[26] Mundim KC, Baraldi S, Machado HG, Vieira FMC (2020). Temperature coefficient (Q10) and its applications in biological systems: Beyond the Arrhenius theory. Ecol. Model..

[27] Clarke, K. G. in *Bioprocess Engineering* (ed Kim Gail Clarke) 75–96 (Woodhead Publishing, 2013).

[28] Pinto GAS, Leite SGF, Terzi SC, Couri S (2001). Selection of tannase-producing *Aspergillus nige*r strains. Braz. J. Microbiol..

[29] Ahmed RF, Hikal MS, Abou-Taleb KA (2020). Biological, chemical and antioxidant activities of different types Kombucha. Ann. Agric. Sci..

[30] Marques MR (2012). An in vitro analysis of the total phenolic content, antioxidant power, physical, physicochemical, and chemical composition of *Terminalia catappa* Linn fruits. Food Sci. Technol..

[31] Singleton VL, Orthofer R, Lamuela-Raventós RM (1999). Methods in Enzymology.

[32] Sen A, Batra A (2012). Determination of antimicrobial potentialities of different solvent extracts of the medicinal plant: *Phyllanthus*
*amarus* Schum. and Thonn. Int. J. Green Pharm..

[33] Singariya P, Kumar P, Mourya KK (2012). Antimicrobial activity of fruit coat (calyx) of *Withania somnifera* against some multi drug resistant microbes. Int. J. Biol. Pharm. Res..

[34] Clinical and laboratory standard Institute. *Antimicrobial susceptibility challenges*. (Versions of M100 and M60, Wayne, PA 19087,, 2015).

[35] Rabe T, Mullholland D, van Staden J (2002). Isolation and identification of antibacterial compounds from Vernonia colorata leaves. J. Ethnopharmacol..

[36] Galal GF, Abd-Elhalim BT, Abou-Taleb KA, Haroun AA, Gamal RF (2021). Toxicity assessment of green synthesized Cu nanoparticles by cell-free extract of *Pseudomonas silesiensis* as antitumor cancer and antimicrobial. Ann. Agric. Sci..

[37] Muijs D (2022). Doing Quantitative Research in Education With IBM SPSS Statistics.

[38] Gupta R, Gigras P, Mohapatra H, Goswami VK, Chauhan B (2003). Microbial α-amylases: A biotechnological perspective. Process Biochem..

[39] Nadaf NH, Ghosh JS (2011). Production, purification and characterization of tannase from *Rhodococcus* NCIM 2891. Curr. Res. J. Biol. Sci..

[40] Ma W-L (2015). Production and partial purification of tannase from Aspergillus ficuum Gim 3.6. Prep. Biochem. Biotechnol..

[41] Chhokar V, Sangwan M, Beniwal V, Nehra K, Nehra KS (2010). Effect of additives on the activity of tannase from *Aspergillus awamori* MTCC9299. Appl. Biochem. Biotechnol..

[42] Viswanath V (2015). Biosynthesis of tannase from cashew testa using *Aspergillus niger* MTCC5889 by solid state fermentation. J. Food Sci. Technol..

[43] Ramos EL (2011). Catalytic and thermodynamic properties of a tannase produced by *Aspergillus niger* GH1 grown on polyurethane foam. Appl. Biochem. Biotechnol..

[44] de Sena AR (2018). Kinetic, thermodynamic parameters and in vitro digestion of tannase from Aspergillus tamarii URM 7115. Chem. Eng. Commun..

[45] Yao J, Guo GS, Ren GH, Liu YH (2014). Production, characterization and applications of tannase. J. Mol. Catal. B Enzym..

[46] Jana A (2013). Structural characterization of thermostable, solvent tolerant, cytosafe tannase from *Bacillus subtilis* PAB2. Biochem. Eng. J..

[47] Vieille C, Zeikus GJ (2001). Hyperthermophilic enzymes: Sources, uses, and molecular mechanisms for thermostability. Microbiol. Mol. Biol. Rev..

[48] Cavalcanti RMF, Martinez MLL, Oliveira WP, Guimarães LHS (2020). Stabilization and application of spray-dried tannase from *Aspergillus fumigatus* CAS21 in the presence of different carriers. 3 Biotech.

[49] Cavalcanti RMF, Jorge JA, Guimarães LHS (2018). Characterization of *Aspergillus fumigatus* CAS-21 tannase with potential for propyl gallate synthesis and treatment of tannery effluent from leather industry. 3 Biotech..

[50] Sabu A, Shegal Kiran G, Pandey A (2005). Purification and characterization of tannin acyl hydrolase from *Aspergillus niger* ATCC 16620. Food Technol. Biotechnol..

[51] Rodríguez-Durán LV, Valdivia-Urdiales B, Contreras-Esquivel JC, Rodríguez-Herrera R, Aguilar CN (2011). Novel strategies for upstream and downstream processing of tannin acyl hydrolase. Enzyme Res..

[52] Battestin V, Macedo GA (2007). Effects of temperature, pH and additives on the activity of tannase produced by Paecilomyces variotii. Electron. J. Biotechnol..

[53] Farias GM, Gorbea C, Elkins JR, Griffin GJ (1994). Purification, characterization, and substrate relationships of the tannase from *Cryphonectria parasitica*. Physiol. Mol. Plant Pathol..

[54] Maalej H, Ben Ayed H, Ghorbel-Bellaaj O, Nasri M, Hmidet N (2014). Production and biochemical characterization of a high maltotetraose (G4) producing amylase from *Pseud**omonas*
*stutzeri* AS22. Biomed. Res. Int..

[55] Rout S, Banerjee R (2006). Production of tannase under mSSF and its application in fruit juice debittering. Indian J. Biotechnol..

[56] Srivastava A, Kar R (2010). Application of immobilized tannase from *Aspergillus niger* for the removal of tannin from myrobalan juice. Indian J. Microbiol..

[57] Selwal MK (2011). Tannase production by *Penicillium atramentosum* KM under SSF and its applications in wine clarification and tea cream solubilization. Braz. J. Microbiol..

[58] Kapoor A, Iqbal H (2013). Efficiency of tannase produced by *Trichoderma Harzianum* MTCC 10841 in pomegranate juice clarification and natural tannin degradation. Int. J. Biotechnol. Mol. Biol. Res..

[59] Nandi, S. & Chatterjee, A. Extraction, partial purification and application of tannase from *Aspergillus niger* MTCC 2425. *Int. J. Food Sci. Nutr.* **1** (2016).

[60] Xu X-Y (2019). Effects of tannase and ultrasound treatment on the bioactive compounds and antioxidant activity of green tea extract. Antioxidants.

[61] Apriyani TW, Supriyadi S, Gunadi R (2022). Improvement of antioxidant activity and sensory quality of Pagilaran’s Tea clones treated by tannase. AgriTECH.

[62] Raghuwanshi S, Misra S, Saxena RK (2013). Enzymatic treatment of black tea (CTC and Kangra orthodox) using *Penicillium charlesii* tannase to improve the quality of tea. J. Food Process. Preserv..

[63] Martins IM, Roberto BS, Blumberg JB, Chen CYO, Macedo GA (2016). Enzymatic biotransformation of polyphenolics increases antioxidant activity of red and white grape pomace. Food Res. Int..

[64] Hidayathulla S, Shahat AA, Alsaid MS, Al-Mishari AA (2018). Optimization of physicochemical parameters of tannase post-purification and its versatile bioactivity. FEMS Microbiol. Lett..

[65] Monteiro, G. *et al.* in *Saccharomyces* (eds T.P. Basso & L.C. Basso) Ch. 2, 17–36 (IntechOpen, 2021).

[66] Shao D (2015). Inhibition of gallic acid on the growth and biofilm Fofrmation of *Escherichia coli* and *Streptococcus mutans*. J. Food Sci..

[67] Kang J, Liu L, Liu M, Wu X, Li J (2018). Antibacterial activity of gallic acid against *Shigella flexneri* and its effect on biofilm formation by repressing mdoH gene expression. Food Control.

[68] Rajamanickam K, Yang J, Sakharkar MK (2019). Gallic acid potentiates the antimicrobial activity of tulathromycin against two key bovine respiratory disease (BRD) causing-pathogens. Front. Pharmacol..

[69] Selvaraj S, Amaral JM, Murty VR (2022). Kinetics and antimicrobial activity of gallic acid by novel bacterial co-culture system using Taguchi’s method and submerged fermentation. Arch. Microbiol..

[70] Borges A, Ferreira C, Saavedra MJ, Simões M (2013). Antibacterial activity and mode of action of ferulic and gallic acids against pathogenic bacteria. Microb. Drug Resist..

[71] Samad MA, Hashim SH, Simarani K, Yaacob JS (2016). Antibacterial properties and effects of fruit chilling and extract storage on antioxidant activity, total phenolic and anthocyanin content of four date palm (*Phoenix dactylifera*) cultivars. Molecules.

[72] Pudlo M, Demougeot C, Girard-Thernier C (2017). Arginase inhibitors: A rational approach over one century. Med. Res. Rev..

[73] Pinho E (2014). Antibacterial potential of northeastern Portugal wild plant extracts and respective phenolic compounds. Biomed. Res. Int..

[74] Anwar R, Hajardhini P (2022). Antibacterial activity of gallic acid from the leaves of *Altingia excelsa* Noronha to *Enterococcus faecalis*. Open Access Maced. J. Med. Sci..

